# Herbal medicine from the perspective of type II diabetic patients and physicians: what is the relationship?

**DOI:** 10.1186/s12906-020-2854-4

**Published:** 2020-02-28

**Authors:** Aljawharah Alqathama, Ghadeer Alluhiabi, Halah Baghdadi, Lujain Aljahani, Ola Khan, Sara Jabal, Shorooq Makkawi, Farah Alhomoud

**Affiliations:** 1 Department of Pharmacognosy, Pharmacy College, Umm Al-Qura University, Makkah, 21955 Saudi Arabia; 2 Department of Clinical Pharmacy and Pharmacy Practice, College of Clinical Pharmacy, Imam Abdulrahman Bin Faisal University, Dammam, Saudi Arabia

**Keywords:** Diabetes, Herbal medicine, Herbs, Patients, Physician, Prevalence, Knowledge, Belief, Attitude, Relationship

## Abstract

**Background:**

Diabetes mellitus (DM) is a major public health problem and one of the most challenging diseases worldwide. According to the World Health Organization (WHO), the Kingdom of Saudi Arabia (KSA) has the second highest rate of diabetes in the Middle East and seventh highest globally. Some diabetic patients may prefer to use alternative approaches such as herbal remedies to control their blood glucose level and this study aims to assess the prevalence of herbal usage and to evaluate users’ and doctors’ knowledge, attitudes and beliefs about herbal medicine as well as the patient/doctor relationship in this regard.

**Method:**

A cross-sectional survey was conducted in several hospitals and medical centres in Makkah, KSA, between January and March 2019. Around 289 type II diabetic patients and 105 doctors were interviewed.

**Results:**

We found that 68% of the participants were frequent consumers of herbal remedies, especially cinnamon, ginger and fenugreek. Patients’ knowledge of herbal usage was mainly gleaned from family and friends as well as social media, and we found that many (71.4%) did not bother to consult or inform their doctors about their choice to self-medicate with herbs. Patients had no concerns regarding the efficacy and safety of herbal usage use in diabetes, as around half of the participants believe that herbal medicine is effective (54%) and safe (46%) for treating symptoms of diabetes. Two-thirds of the doctors (66%) routinely ask patients whether they use herbs for their condition. Although 25% of the doctors took a positive view of herbal medicine in relation to diabetes, others expressed concerns with the rise in herb use and want to see more attention paid to safety aspects.

**Conclusion:**

The study concludes that herbal remedies are commonly used by diabetic patients and that a gap exists in the relationship between patients and doctors concerning the disclosure of herbal remedy use and views on its safety.

## Background

Herbal medicine is a form of healthcare that comes under the category of complementary and alternative medicine (CAM) [1]. As defined by the US National Center for Complementary and Alternative Medicine, CAM is a group of heterogeneous medical and healthcare systems, substances, supplements and procedures that are not part of mainstream or conventional medicine [2] Any products originating from plants and consumed or applied to treat illness or maintain health belong to herbal medicine, which is part of CAM practice. It is one of the oldest documented forms of traditional medicine and has been found to be 5000 years old. However, scientific evidence is still lacking for its effectiveness [3].

In most countries, herbal medicines are commonly used among all healthcare systems [4]. According to the World Health Organization (WHO), in developing countries approximately 80% of the population have been found to be using this form of medicine for primary care such as those in Africa and Asia [5]. Similarly, nowadays herbal medicines are also in great demand in the developed world as interest in herbal medicine has soared over the last two decades [6]. In Europe, and the United States (US), around 75% (France) and 42% of the population respectively, were found to use herbal medicine at least once [7, 8]. Similar numbers have been seen in Middle Eastern countries such as Egypt (86%) and the Kingdom of Saudi Arabia (KSA) (88%). Therefore, herbal medicines have achieved high academic, industrial and economic interest due to their global prevalence [9, 10]. CAM is also common in KSA, where herbs, honey, dietary products, prayer and cupping are widely used for medicinal purposes by the general public. Most of the existing studies were conducted in the Riyadh region, with very few in other areas, such as the west of KSA, which is known for its cultural diversity and would be a useful area for research [11].

Diabetes mellitus is among the most prevalent chronic diseases and is associated with high rates of morbidity and mortality [12]. WHO estimated that 347 million people worldwide were diagnosed with diabetes in 2013 and this figure is expected to double by 2030 [13]. Recently, WHO estimated 1.6 million deaths were directly linked to diabetes [14]. The majority of those who are affected are the middle-income populations living in Asia, Africa and South America [13]. Diabetes causes one death every 6 sec with death cases (5 million), a rate higher than all deaths from human immunodeficiency virus (HIV) (1.5 million), tuberculosis (1.5 million) and malaria (0.6 million) combined. If left untreated, diabetes can lead to potential complications that include damage in organs such as the kidneys, heart, eyes, blood vessels and nerves [15].

Emerging data show that diabetic patients use CAM with varying degrees of success. About 2 to 3.6 million people use CAM in the USA to manage diabetic symptoms [13]. A Canadian study found that 44% of diabetic patients used over-the-counter supplements, compared to 31% patients who used alternative medication [16]. Similarly, in Australia it was found that 25% of diabetic patients had been using CAM in the 5 years between 2011 and 2015 [16]. In the US, herbal medicine use has increased by up to 380% [13]. Previous studies have revealed that diabetics tend to use herbal remedies to minimize dissatisfaction with conventional therapies. Another reason for using herbs is their concern about possible side effects caused by conventional therapies. This is because these patients believe that herbal remedies from natural sources are safer and more effective [15].

Past studies indicate that the beliefs and attitudes of healthcare providers about CAM vary greatly, from scepticism to strong support [17]. The literature review shows that healthcare professionals often demonstrate minimal formal education and little real knowledge of alternative medicine, and that there exists a significant gap between the high use of CAM by patients and acknowledgement or acceptance by healthcare professionals. Studies show that patients are reluctant to discuss their use of CAM with healthcare professionals [17, 18]. With regard to diabetes treatment, there is a lack of studies investigating the patient/doctor relationship, as well as the attitudes and beliefs of healthcare professionals regarding herbal medicine in diabetic management. Therefore, the purpose of this study was to investigate the knowledge, attitudes and beliefs of diabetic patients and their physicians regarding herbal usage, as well as the nature of the patient/doctor relationship in terms of discussing this practice with patients.

## Methods

### Study design and setting

A descriptive, cross-sectional study design using structured predesigned questionnaires which were distributed among type II diabetic outpatients and doctors who are working or registered at all four government hospitals (Al Noor, Heraa, King Abdulaziz and King Faisal hospitals) and medical centres (13 centres) in Makkah, Saudi Arabia, was conducted for the period from January to March 2019.

### Sampling and recruitment strategies

#### Justification of the chosen medical centres and representativeness

A purposive sample was selected; all government hospitals registered at the Ministry of Health in Makkah Province which have endocrinology clinics were involved. Another sampling approach was also considered; a convenience sampling technique based on locating endocrinology clinics in medical centres distributed in several locations in Makkah (12 medical centres) was carried out.

#### Sample size and recruiting patients and doctors

For patients, all ≥18 years old patients diagnosed with type II diabetes, not pregnant and who were able to take part were included and interviewed during their visits for follow-up care at endocrinology clinics/medical centres or for refill medications. The doctors in the sample included all endocrinologists who treated diabetic patients at hospitals where there was a department of Internal Medicine or an endocrinology clinic as well as doctors dealing with and giving follow-up treatment to diabetic patients in medical centres. Doctors in the Internal Medical department who do not deal with diabetic patients were excluded. Only diabetic type II patients and their doctors were verbally asked to participate after the study purpose was explained and they were provided with informed consent forms to sign before participation. The sample size was calculated using an online sample calculator (Raosoft). A single population proportion formula was used with the assumption of 95% confidence interval, 5% margin of error, among 3,852,000 diabetic patients in KSA as it was estimated by the International Diabetes Federation (IDF) [19]. Therefore, a total of 385 participants were required. However, due to time limitations in the sample hospitals, only 309 patients took part in this project. Due to the fact that it was difficult to obtain the number of registered endocrinologists in Internal Medicine departments and endocrinology clinics in the MOH in Makkah, the sample size was justified by the power of the study and finally adjusted for non-response/unintended error using the following statistical formula [20].

$$ n=\frac{{\left({Z}_{1-\beta}\right)}^2\left[p\left(1-p\right)\right]}{E^2} $$

A sample size of 105 can achieve more than 80% power with 20% non-response/unintended error rate.

### Study instrument

A literature review was undertaken to develop both questionnaires to assess the prevalence of herbal usage and evaluate the knowledge, attitudes and beliefs of diabetic patients and their physicians regarding herbal medicine. After drafting the initial version of each questionnaire, the advisory group (i.e. pharmacy academic staff) were given these questionnaires to comment on the wording and the content, as per the requirements of the quality assessment. Comments and feedback were obtained and the final questionnaire was developed subsequently. The questionnaire was then piloted to five randomly chosen diabetic patients in one of the endocrinology clinics for feedback on wording, understanding, and ease of use. This helped to assess the feasibility of the questionnaire and acted as a method of face validity. Data collected from the pilot study were not included in the final analysis.

The questionnaires were constructed in several parts and covered knowledge, attitudes and beliefs regarding herbal usage. The patients’ questionnaire was divided into two sections, the first focusing on patient-related information such as age, gender, living area, education level and presence of comorbidities, and the second investigated the prevalence of herbal product usage, knowledge of correct usage and disclosure of usage to their doctors. The questionnaire was conducted in Arabic for the patients’ convenience. The doctors’ questionnaire covered areas such as experience of and familiarity with herbal medicine, outcomes, attitudes to herbal remedies and how they deal with patients who use herbs.

In addition, to ensure reliability, which depends highly on the reproducibility of the collected data, we collected data using identical techniques at the same time to result in the same findings as mentioned previously [21]. To ensure this, steps were taken to follow the protocol for data collection. Questionnaires were distributed by the researchers, who were trained to interview participants at the diabetic clinics, to ensure they were received by the target populations. Moreover, data collection was performed on one single occasion and the collected data from all involved hospitals were subsequently analysed. To reduce the inadvertent bias in interpreting responses from open-ended questions, closed questions were chosen.

### Data processing and analysis

The completed survey was processed and analysed using a quantitative procedure conducted using the statistical methods available in the Statistical Package for the Social Sciences (SPSS 26 software). The descriptive statistics were generated and possible relationships between different variables were assessed using a chi-square test.

### Ethical approval

The study was approved by the Institutional Review Board (IRB) of the Ministry of Health, IRB number H-02-K076–1811-063 on 12th December 2018. Written informed consent was obtained from all participants as stipulated by the IRB Committee.

## Results

### Sample of type II diabetic patients

**Fig. 1:**
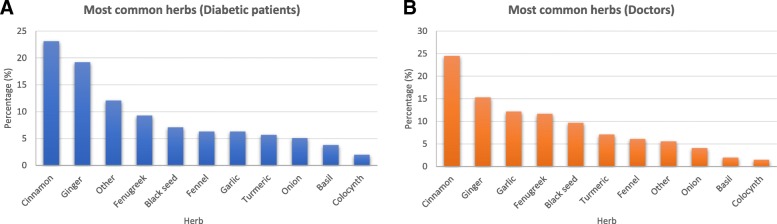
The most frequently used herbs from the perspective of the diabetic population (**a**) and their doctors (**b**)

A total of 309 questionnaires were distributed among diabetic patients; 298 completed the questionnaires, resulting in 298 responses (response rate = 96%). The data show that 203 (68.1%) of the diabetic participants used herbal medicines to manage their condition. Of those 188, (92.6%) have comorbidities including hypo/hypertension, cardiovascular diseases and others (see Table 1). The most commonly used herbs were cinnamon (23.1%), ginger (19.2%), fenugreek (9.3%) and others such as garlic, onions, basil, black seed, fennel, peppermint, anise, cumin and parsley (Fig. 1). More than half of the participants stated that they used the herbs singly rather than as part of a mixture. These herbs listed in the study questionnaire are widely available in KSA and are well known to help in managing type II diabetes.

**Table 1:** Demographics and comorbidities of type II diabetic patients

Variables	Overall (n, %)	Herbal medicine use (n)	
		Yes (*n* = 203)	No (*n* = 95)
Sex			
Male	128 (43)	80	48
Female	170 (57)	123	47
Age			
18–45	44 (14.8%)	31	131
46–60	153 (51.3%)	112	41
61–75	90 (30.2%)	54	36
More than 75	11 (3.7%)	6	5
Educational status			
Illiterate	100 (33.6%)	65	35
Primary	77 (25.8%)	56	21
Intermediate	35 (11.7%)	24	11
High school	35 (11.7%)	23	12
University/college	51 (17.1%)	35	16
Comorbidities			
Hypo/hypertension	155 (55.8%)	107	48
Heart disease	47 (16.9%)	32	15
Other	76 (27.3%)	49	27
Duration of disease			
Less than 1 year	11 (3.7)	7	4
1–5 year	63 (21.1)	40	23
6–9 year	56 (18.8)	44	12
10 years and more	168 (56.4)	112	56

As shown in Table 1, it was found that more than half of herbal users were women (*n* = 123; 60.5%) and aged between 46 and 60 years (*n* = 112; 37.5%). The relationship between education and herbal use was measured, and it was found that 31.1% of herbal users had not completed their education and that 26.6% had received only primary education (Table 1). Statistical analysis was conducted using chi-square test; none of the patients’ characteristics have shown a significant relation to the herbal consumption.

Those who were diagnosed with diabetes for over 10 years (*n* = 168; 56.4%) commonly utilized herbs for such conditions at least once a day (*n* = 112, 55.1%). In terms of herb preparation methods, boiling the herbs (decoction) was the most common method of preparation, cited by approximately half of the herbal users (*n* = 98; 40.7%), followed by soaking the herbs (*n* = 74, 30.7%) and then either using the plant parts intact or as a powder (not shown in table).

Assessing patient sources of information (Table 2) about efficacy of herbs revealed that recommendation by family and friends was the most common (*n* = 156; 50.6%), followed by social media (*n* = 73; 23.7%) and websites (*n* = 49; 15.9%).

**Table 2:** Source of herbal medicine information among herbal users

Source of information	Number	Percentage
Family and friends	156	50.6%
Social media	73	23.7%
Healthcare providers’ recommendation	30	9.7%
Websites (internet)	49	15.9%

Patients’ beliefs about the efficacy and safety of herbal remedies are illustrated in Table 3. Data show that more than one-half of the sample (54.7%) either believe or strongly believe that herbal remedies are effective. In addition, only around 28% of the survey participants disagreed with the statement that herbs do not interact with their orthodox medication. There was a statistical significant relationship between the continuous use of herbs and the beliefs that the herbs would not interact with diabetic medication and that they would have a positive effect on controlling blood glucose levels (*p* < 0.001, *p*-value not shown in the table).

**Table 3:** Diabetic patients’ knowledge and beliefs about herbal usage

Statement	Strongly Agree	Agree	Neutral	Disagree	Strongly Disagree
Patients believe that herbs have a good effect	89 (29.9%)	74 (24.8%)	70 (23.5%)	24 (8.1%)	41 (13.8%)
Patients believe that herbs are safe	81 (27.2%)	57 (19.1%)	93 (31.2%)	33 (11.1%)	34 (11.4%)
Patients believe that herbs do not interact with diabetic medications	30 (10.1%)	35 (11.7%)	148 (49.7%)	52 (17.4%)	33 (11.1%)
Patients believe that it is possible to use herbs only	7 (2.3%)	18 (6.0%)	42 (14.1%)	60 (20.1%)	171 (57.4%)

The participants were asked whether they discussed their herbal usage with their doctors and it was found that around 145 participants (71.4%) (Table 4) did not consult their doctors about using herbs to control blood glucose levels. As shown in Table 4, being afraid of their doctor’s disagreement or negative response was the least mentioned reason (*n* = 16; 11.0%). The main reason for non-disclosure of herb use by diabetic patients (*n* = 57; 39.3%) was not fear of disapproval by the medical establishment, but the fact that doctors rarely ask patients about their CAM use. A significant number of participants (*n* = 72; 49.6%) believed that doctors would not support herbal usage because of possible interactions with anti-diabetic medication, or the lack of reliable sources supporting herb efficacy (Table 4).

**Table 4:** Patients’ ability to disclose herbal usage to their doctors

Have you told or consulted your doctor about using these herbs	Number	Percentage
Yes	58	28.6%
No	145	71.4%
**Reasons**		
For not telling your doctor		
The doctor didn’t ask me	57	39.3%
It is not necessary to inform the doctor about the herbs I use	51	35.1%
Other	21	14.4%
Possible doctor disagreement about herbal usage	16	11.0%
Why doctors do not support herbal usage		
I do not know	47	32.4%
These plants may react with drugs and affect their effectiveness	45	31.0%
Lack of reliable scientific studies	27	18.6%
Herbs are not effective for diabetic management	14	9.6%
The difficulty in using herbs for diabetic management	7	4.8%
Other	5	3.4%

### Sample of physicians

**Fig. 2:**
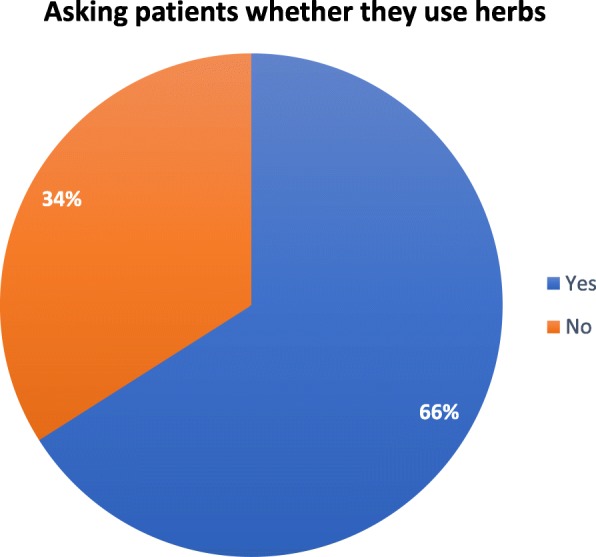
Doctors’ knowledge about herbal usage by diabetic patients

Of the 106 recruited doctors, 105 completed the questionnaire, representing a 99% response rate. The results show that 70 doctors (66%) routinely asked their patients whether they were also using traditional herbal medicine alongside conventional medication. The majority of these doctors were fairly newly qualified and had less than 5 years’ experience (Fig. 2).

Data illustrate that most of doctors (*n* = 74; 70.5%) (Table 5) believe that only a small percentage of diabetic patients are herbal users (0–25%), but this contrasts with data obtained from the patients’ category, which show significantly higher actual herbal use (68.1%). The findings show that 37.1% of the doctors believed that herbal medicine had no effect in regulating blood glucose levels. However, 25.7% (*n* = 27) indicated a belief that herbs could have a positive effect on blood glucose, while the rest were of the opinion that herbs could produce a negative effect by either reducing or elevating sugar levels. According to the doctors in the sample, the most commonly used herbs were cinnamon, ginger, garlic and fenugreek, which was in agreement with patient data.

**Table 5:** Doctors’ knowledge about the percentage of diabetic herbal users and effect of herbs on them

What is the percentage of herbal users?	Number	Percentage
0–25	74	70.5%
26–50	11	10.5%
51–75	1	1.0%
76–100	19	18.1%
**What is the effect of herbs on blood glucose level**		
No effect	39	37.1%
Positive effect by improving glucose level	27	25.7%
Negative effect by raising glucose level	8	7.6%
Negative effect by reducing glucose level	8	7.6%

Results analysis reveals that about 75 (71.4%) of the doctors agree and strongly agree that patients used herbs to improve blood glucose levels, as well as other purposes such as treating other diseases (*n* = 59; 56.2%) and weight loss (*n* = 44; 41.9%) (Table 6). In addition, data show that 64 (61%) and 69 (65.7%) of the doctors agree and strongly agree that they regularly asked their patients about herbal use in order to avoid herb side effects and herb-drug interactions with conventional treatment, respectively. It was also found that a high proportion of participants strongly believed that not all herbs are safe (n = 59; 56.2%). Furthermore, only a quarter of the doctors (*n* = 28; 26.7%) in the study used information based on scientific reports about herb uses in the practice, indicating that healthcare professionals should be encouraged to refer to scientifically reliable sources for their information on herbal medicine.

**Table 6:** Doctors’ beliefs and attitudes towards herbal medicine

Statement	Strongly Agree	Agree	Neutral	Disagree	Strongly Disagree
Patients use herbs to improve blood glucose level	21 (20.0%)	54 (51.4%)	23 (21.0%)	6 (5.7%)	1 (1.0%)
Patients use herbs to treat other disease	13 (12.4%)	46 (43.8%)	37 (35.2%)	7 (6.7%)	2 (1.9%)
Patients use herbs to lose weight	8 (7.6%)	36 (34.3%)	38 (36.2%)	19 (18.1%)	4 (3.8%)
I ask patients about herbal usage to avoid side effects	26 (24.8%)	38 (36.2%)	26 (24.8%)	11 (10.5%)	4 (3.8%)
I ask patients about herbal usage to avoid herb-drug interaction	29 (27.6%)	40 (38.1%)	23 (21.9%)	10 (9.5%)	3 (2.9%)
Not all herbs are safe for diabetic patients	59 (56.2%)	26 (24.8%)	12 (11.4%)	3 (2.9%)	5 (4.8%)
I recommend herbs for diabetic patients based on scientific information	7 (6.7%)	21 (20.0%)	39 (37.1%)	22 (21.0%)	16 (15.2%)
I recommend herbs for diabetic patients based on patients’ previous experiences	2 (1.9%)	11 (10.5%)	31 (29.5%)	35 (33.3%)	26 (24.8%)
Doctor’s awareness about herbal usage reflects positively on patient’s health	27 (25.7%)	34 (32.4%)	21 (20.0%)	15 (14.3%)	8 (7.6%)
I would refer diabetic patients to a herbal medicine clinic if available	13 (12.4%)	20 (19.0%)	36 (34.3%)	25 (23.8%)	11 (10.5%)

As shown in Table 6, 61 (58.1%) of the doctors agreed that their awareness of herbal usage would have an effect on patient health. The study participants were asked whether they would refer their patients to a herbal medicine clinic if this was made available. More than a third of the participants (*n* = 36, 34.3%) said they would not refer patients.

## Discussion

Data revealed that two-thirds of the sample who suffer from diabetes were frequently using alternative therapies such as herbal medicine to control their glucose level and improve their health, in accordance with two previous studies conducted in Africa (Ethiopia, Nigeria) [22, 23]. However, two other studies conducted in Asia (Thailand) and the Middle East (KSA and UAE) showed that less than one-third of the sample were found to be using herbal medicine [24–26]. The differences between these numbers could be due to the discrepancy in the nature of these studies regarding the geographical location, levels of the population’s dependency on herbal remedies, and because some of these studies included all the traditional CAM practices. This variability was also reported in a review mentioned in the literature of studies from nine countries, where studies of diabetic populations revealed a prevalence of alternative medicine use ranging from 17 to 72%; however, herbal medicine is still an important feature of diabetes management worldwide [27]. Cinnamon and fenugreek were the most commonly used herbs not only in the KSA but also in other Arabic countries such as Bahrain, Oman, Iraq and Jordan [24]. This is because they are widely available in these countries due to the fact that all of these herbs thrive in their similar climates [28]. Similarly, in the USA and Canada, cinnamon was commonly used as well. In contrast, in Malaysia, bitter gourd and misai kucing were the most commonly used herbs; this difference is probably due to the differences in culture and geographical environment [29].

Older people (aged between 46 and 60) tend to take more herbs than younger people because they are more likely to seek alternative treatment options [22]. Similarly, in Alrowais’ cross-sectional study which was conducted in four major hospitals in Riyadh, it was reported that patients aged between 60 and 75 years were the most frequent users of herbs for diabetic management [30]. This study agrees with the findings of Ching, Amaeze and Mekuria’s studies related to gender differences, which showed that women were more likely to consume herbal remedies than men [22, 23, 29]. In a study by Shih et al. in Taiwan, patients aged 20–69 years traditional Chinese medicine use frequency was significantly higher in women than in men across all age groups. According to their study, this is because women in these societies are the primary decision makers in family healthcare, and are more open to alternatives, tending to support CAM services more than men [31]. It was found here that 31% of herbal users had not completed their education and that 26.6% had only completed primary education. In Ilhan’s study, which was conducted among herbal medicine users in Turkey, it was found that 58% of the users had received only primary education [32]. However, Alalami’s study, conducted in the UAE, found that more highly educated patients (secondary and tertiary levels) were the most frequent users of herbs [25].

Regarding the source of information used by patients to obtain information on herbs, it was shown that the majority of participants in this study relied on their families and friends (50.6%), social media (23.7%) and the internet (15.9%) as the main source of information on herbs used for diabetes. A study in Australia of pregnant women using CAM practice reported that nonprofessional sources of information were found to be influential in the studied population [33]. However, healthcare providers were the least likely source to be used, which was in line with one previous study conducted in Ethiopia [23].

Our study findings were in line with one previous study exploring the knowledge, attitude and practice (KAP) of CAM therapies among patients with type II diabetes [34]; both studies showed that more than one-half of the participants believed that herbal medicine products have remarkably higher efficacy in controlling blood glucose levels and a wider safety window. This could be an indicator for potential plant toxicity unawareness by users that might be caused by improper use or handling. Half of the diabetic patients in our study did not consult their doctors before starting to use herbal remedies, which was consistent with another previous study which reported that 68% of participants had not disclosed their herbal usage to their doctors [35]. Reasons reported by previous studies for such lack of disclosure were: doctors rarely ask patients about their herbal use, as reported here [36], or due to an anticipated negative response [23]. The absence of clear communication between patients and healthcare providers may have a potentially serious effect on patient health and outcomes as patients may experience plant toxicity due to improper use, expected and unexpected side effects, and herb-drug interaction with hypoglycaemic drugs.

Doctors in our study believed that the majority of patients are non-herb users, but this is not consistent with patient response. This indicates the presence of gap in communication between patients and doctors. Solutions such as educational intervention may be effective, whereby doctors would be adequately equipped to communicate with their patients about herbs for specific conditions. Other solutions include continuing education programmes, the availability of reputable references such as pharmacopoeias, placing books on herbal medicine in hospital libraries and integrating herbal medicine studies into medical school curricula. Furthermore, patient education via social media or medical staff and the availability of integrative clinics in hospitals would encourage patients to discuss their herbal use with doctors. These measures may all help in narrowing the communication and knowledge gap.

Furthermore, there was no significant relation between physician’s characteristics and the likelihood of recommending herbal usage to patients. A study conducted in the USA evaluating the attitudes of physicians at an academic medical centre towards CAM, reported that physicians under 46 years were more inclined to accept its potential value and 30% believed that knowledge of CAM practices would lead to better patient outcomes [37]. In a study conducted in the USA to determine general attitudes and approaches to CAM among obstetric and gynaecology patients and physicians, the latter declared that they need to be aware of recent updates about herbal medicine as they are relevant to patient health [36]. Furthermore, half of the participants in our study were neutral or against referring patients to a herbal medicine clinic if one is available, which is different to a study in the USA indicating that healthcare providers are more likely to refer patients to CAM or herbal medicine practitioners [18].

### Strength and limitations

One of the strengths of this study is that it investigates both diabetic patients and their doctors at the same time to illustrate clearly the relationship between them and clarify the current situation regarding the use of herbal medicine for diabetes. The study has certain limitations including generalization from a convenient sample size that was collected from hospitals in one region. However, these findings should not be generalized to the wider population. In addition, the interview-based questionnaire, the method of collecting data from the diabetic patients, may have resulted in interview bias, although it is accepted as a legitimate scientific method. However, most of the patients would not have been able to fill in a self-administered questionnaire as they suffer from complications due to their disease. Regarding healthcare professionals, it was not possible to obtain the exact number of physicians in KSA to calculate sample size precisely and they were the only category included in this study, but broadening the sample to include other healthcare providers such as pharmacists and nurses would make it representative for all healthcare professionals.

## Conclusion

This research revealed that managing blood glucose levels with herbal medicine is common practice among diabetic patients and many patients did not disclose this to their doctors, indicating a gap or miscommunication in their relationship which has an impact on patient health. Thus, doctors need to ask patients about herbal usage, encourage them to talk about it, and be aware of the most recent updates in this rapidly changing field in order to be able to give sound advice on the proper use of herbs for diabetes management. In addition, perception about herb effectiveness and safety is high, suggesting that the general public needs to be educated about the possible harms caused by self-medicating with herbs. The Ministry of Health as well as individual healthcare providers need to consider how current media channels may be used for this type of educational purpose. More government-based research and educational programmes about herbs for diabetes are needed in KSA to maximize the benefits and minimize the side effects.

## Data Availability

The data used and analyzed during in this study are available from the corresponding author on reasonable request.

## Abbreviations

CAM: Complementary and alternative medicine
IDF: International Diabetes Federation
IRB: Institutional Review Board
KAP: Knowledge, attitude and practice
WHO: World Health Organization
